# Epigenetic Impacts of Non‐Coding Mutations Deciphered Through Pre‐Trained DNA Language Model at Single‐Cell Resolution

**DOI:** 10.1002/advs.202413571

**Published:** 2025-01-30

**Authors:** Zhe Liu, An Gu, Yihang Bao, Guan Ning Lin

**Affiliations:** ^1^ Shanghai Mental Health Center, Shanghai Jiao Tong University School of Medicine, School of Biomedical Engineering Shanghai Jiao Tong University Shanghai 200230 China; ^2^ Shanghai Key Laboratory of Psychotic Disorders Shanghai 200230 China; ^3^ Engineering Research Center of Digital Medicine of the Ministry of Education Shanghai 200230 China

**Keywords:** deep learning, DNA methylation, non‐coding mutations, single‐cell resolution, SNP‐CpG interactions

## Abstract

DNA methylation plays a critical role in gene regulation, affecting cellular differentiation and disease progression, particularly in non‐coding regions. However, predicting the epigenetic consequences of non‐coding mutations at single‐cell resolution remains a challenge. Existing tools have limited prediction capacity and struggle to capture dynamic, cell‐type‐specific regulatory changes that are crucial for understanding disease mechanisms. Here, Methven, a deep learning framework designed is presented to predict the effects of non‐coding mutations on DNA methylation at single‐cell resolution. Methven integrates DNA sequence with single‐cell ATAC‐seq data and models SNP‐CpG interactions over 100 kbp genomic distances. By using a divide‐and‐conquer approach, Methven accurately predicts both short‐ and long‐range regulatory interactions and leverages the pre‐trained DNA language model for enhanced precision in classification and regression tasks. Methven outperforms existing methods and demonstrates robust generalizability to monocyte datasets. Importantly, it identifies CpG sites associated with rheumatoid arthritis, revealing key pathways involved in immune regulation and disease progression. Methven's ability to detect progressive epigenetic changes provides crucial insights into gene regulation in complex diseases. These findings demonstrate Methven's potential as a powerful tool for basic research and clinical applications, advancing this understanding of non‐coding mutations and their role in disease, while offering new opportunities for personalized medicine.

## Graphical abstract

Methven is a deep‐learning framework that predicts the impact of non‐coding SNPs on DNA methylation at single‐cell resolution. By integrating pre‐trained DNA language model with single‐cell ATAC‐seq data, Methven models SNP‐CpG interactions across genomic distances up to 100 kbp. It supports both classification and regression outputs, enabling dynamic predictions of the direction and magnitude of methylation changes, and uncovers cell‐type‐specific mechanisms linked to diseases such as rheumatoid arthritis.

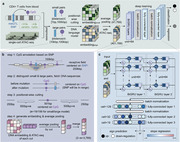

## 1 Introduction

DNA methylation is a key epigenetic modification essential for regulating gene expression, with critical roles in cellular differentiation and disease pathogenesis.^[^ ^1^, ^2^, ^3^ ^]^ This modification is embedded within a complex regulatory network, influenced by non‐coding regions such as enhancers and silencers, which mediate chromatin structure, DNA accessibility, and protein interactions.^[^ ^4^, ^5^, ^6^ ^]^ Mutations in these non‐coding regions can disrupt chromatin loops or transcriptional machinery recruitment, leading to aberrant methylation patterns associated with diseases such as cancer and autoimmune disorders.^[^ ^7^, ^8^, ^9^ ^]^ For instance, mutations in enhancer regions have been implicated in oncogene activation, while mutations linked to autoimmune diseases may alter the regulation of immune‐related genes.^[^ ^10^, ^11^, ^12^ ^]^ Non‐coding mutations often act synergistically with other genetic or epigenetic modifications, amplifying disease progression.^[^ ^13^ ^]^ Thus, understanding how non‐coding mutations affect these mechanisms is crucial for building accurate models of gene regulation.

Despite significant advances in identifying genetic variants, traditional approaches have primarily focused on direct genomic signals such as transcription factor binding and histone modifications.^[^ ^14^, ^15^, ^16^ ^]^ While these approaches have provided valuable insights, they often overlook the regulatory effects mediated by DNA methylation, especially in non‐coding regions. This limitation hampers our understanding of the broader epigenetic landscape, particularly regarding the long‐range interactions that are critical for proper gene expression and cellular function.^[^ ^17^ ^]^

Moreover, DNA methylation, particularly at CpG sites, is known to be cell type‐specific, making it imperative to study this modification at the single‐cell level.^[^ ^18^, ^19^, ^20^ ^]^ Advances in single‐cell technologies have dramatically improved our ability to map methylation dynamics, uncovering regulatory mechanisms that were previously masked in bulk assays.^[^ ^21^, ^22^ ^]^ The ability to distinguish methylation differences at the single‐cell level is crucial for understanding disease pathology and for developing more precise therapeutic strategies.^[^ ^23^ ^]^ Aberrant methylation patterns in distance cell populations have been linked to a wide spectrum of diseases, including cancer, autoimmune disorders, and neurodevelopmental disorders.^[^ ^23^, ^24^, ^25^ ^]^

However, despite the growing recognition of non‐coding variants and their role in disease, translating this knowledge into methods that predict methylation changes at the single‐cell level remains a challenge.^[^ ^26^ ^]^ Current tools, such as CpGenie,^[^ ^27^ ^]^ were designed to predict the impact of non‐coding variants on DNA methylation but have limitations due to their narrow receptive field of 500 base pairs (bp), hindering their utility to capture regulatory interactions over broader genomic regions. While effective for local SNP‐CpG interactions, CpGenie struggles with more long‐range interactions, such as those involving enhancers located tens of kilobases from their target genes. These long‐range interactions are critical for complex diseases like cancer, where distal enhancer elements play a pivotal role in gene regulation. Moreover, CpGenie is not designed for single‐cell resolution, limiting its ability to capture cell‐specific regulatory changes.^[^ ^27^ ^]^

Other models, such as DeepSea^[^ ^28^ ^]^ and Enformer,^[^ ^29^ ^]^ offer broader receptive fields and can annotate DNA sequences for functional impacts across larger genomic regions. However, these models generate static predictions, lacking the flexibility to account for the dynamic nature of epigenetic regulation. In diseases such as cancer, where regulatory regions like enhancers and promoters undergo temporal changes, these models fall short of capturing the evolving regulatory landscape. Similarly, in autoimmune diseases, where immune cells dynamically respond to environmental stimuli,^[^ ^30^ ^]^ these models struggle to predict context‐specific methylation changes. Furthermore, these models could not explain transcriptome variability between individuals, as they focus on general gene regulation without providing the cell‐type specificity needed to understand how non‐coding variants influence distinct cellular environments.^[^ ^31^, ^32^ ^]^ For instance, in neurodevelopmental disorders, where methylation patterns vary between neuronal subtypes, the lack of single‐cell data integration significantly reduces the accuracy of these models in predicting cell‐specific regulatory changes.^[^ ^28^, ^29^, ^33^ ^]^

To address these limitations, we developed Methven, a deep learning framework designed to predict the effects of non‐coding mutations on DNA methylation at single‐cell resolution. Methven integrates DNA sequences with single‐cell ATAC‐seq data, employing a divide‐and‐conquer strategy to model SNP‐CpG interactions across genomic distances of up to 100 kbp. By supporting both classification and regression tasks, Methven aims to provide more accurate and comprehensive predictions of methylation dynamics, particularly for long‐range regulatory interactions. This framework addresses the gaps in existing models and offers potential applications in understanding the epigenetic underpinnings of complex diseases and advancing personalized medicine.

## 2 Results

### 2.1 Overview of Methven

Methven was designed to predict the impact of non‐coding mutations on methylation sites within a 100 kbp range around single‐nucleotide polymorphism (SNP), specifically at single‐cell resolution. To achieve this, we collected 244,491 cis‐methylation quantitative trait loci (meQTL) data^[^ ^34^ ^]^ from the meQTL EPIC Database,^[^ ^35^ ^]^ specifically focusing on CD4+ T cells (Table S1, Supporting Information). These data were selected for their high‐quality annotation of SNP‐CpG interactions, which renders them well‐suited for modeling methylation changes across a broad range of genomic distances. The CD4+ T cell type was specifically chosen due to its pivotal role in immune regulation and frequent involvement in autoimmune diseases, positioning it as a valuable model for investigating methylation dynamics in disease contexts.

In addition to meQTL data, the corresponding single‐cell ATAC‐seq data from the EpiMap Repository^[^ ^36^ ^]^ were incorporated. The use of single‐cell ATAC‐seq data ensures that Methven can capture chromatin accessibility at the single‐cell level, which is essential for understanding the regulatory dynamics of gene expression and methylation. This single‐cell resolution is crucial for making predictions about cell‐type‐specific methylation patterns that are often masked in bulk assays, enabling a more precise understanding of epigenetic changes in various disease states. By incorporating both meQTL and ATAC‐seq data, Methven mitigates model bias from false‐negative SNPs (i.e., SNPs that affect CpG sites but are not statistically captured), while ensuring that the model retains its generalizability and adaptability across other cell types or tissues.

**Figure 1:**
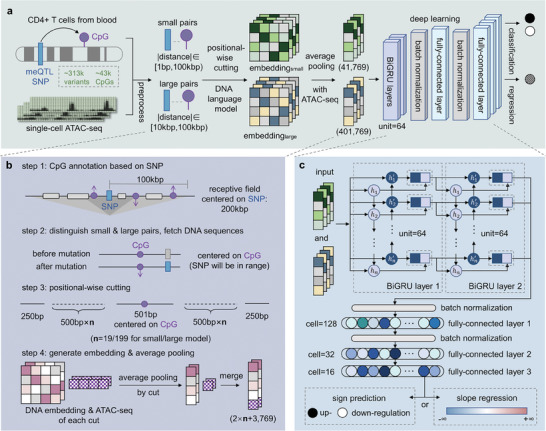
Overview of Methven. a) Prediction of mutation impacts on methylation using DNA sequences and single‐cell ATAC‐seq data. In the data collection phase, meQTL data from CD4+ T cells were used to label the impact of non‐coding mutations on CpG sites. A divide‐and‐conquer strategy was employed, segmenting the dataset into small pairs and large pairs based on the distance between SNPs and CpG sites. DNA sequences after positional‐wise cutting were fed into a DNA language model. The generated DNA embeddings and the corresponding ATAC‐seq data were averaged and concatenated within each cut unit. The concatenated embeddings were finally fed into the deep learning model designed to perform both classification and regression tasks. b) Illustration of the preprocessing pipeline. For each SNP, CpG sites within 100 kbp upstream and downstream were identified based on methylation changes. SNP‐CpG pairs within 10 kbp formed the small dataset, while those between 10 kbp and 100 kbp formed the large dataset. DNA sequences and corresponding ATAC‐seq data were extracted with the CpG site centered. The sequences, both pre‐ and post‐mutation, were then positionally cut around the CpG site, and input into a DNA language model to obtain embeddings. Finally, the DNA embeddings from each cut and the ATAC‐seq data were average pooled and concatenated. c) Details of the deep learning module. The concatenated embeddings are fed into two stacked Bidirectional Gated Recurrent Unit (BiGRU) modules, followed by batch normalization layers and fully connected layers. The classification and regression tasks are handled separately: the classification task predicts the direction of the SNP's impact on CpG methylation levels (upregulation/downregulation), while the regression task estimates the magnitude of this impact (slope).

Methven's architecture comprises two core components (**Figure** **1**): (1) preprocessing of labeled SNP‐CpG pairs to generate embeddings suitable for training, and (2) a deep learning module designed to perform both classification and regression tasks. During preprocessing, CpG sites within a 100 kbp range around each SNP were annotated, and a comprehensive dataset comprising 50190 SNP‐CpG pairs was constructed (Figure 1b). Recognizing that SNPs close to CpG sites may exert more direct and stronger effects, while those at greater distances might involve more complex long‐range regulatory mechanisms, we applied a divide‐and‐conquer strategy. The dataset was split into small pairs (distance <10 kbp, 19,874 pairs) and large pairs (distance between 10kbp and 100kbp, 30,316 pairs). Independent models were trained for each subset, with the Methven‐small model targeting small pairs, and the Methven‐large model focusing on large pairs, allowing each model to capture the specific features relevant to their respective distances.

To enhance the representation of DNA sequences, we selected DNABert2^[^ ^37^ ^]^ as the language model for generating pre‐trained DNA embeddings. We selected DNABert2 due to its efficient Byte Pair Encoding (BPE) tokenization, which improves computational efficiency and captures complex genomic patterns.^[^ ^37^ ^]^ Due to the input sequence length limitations of DNABert2, we implemented a positional‐wise cutting strategy, ensuring that each segment remains central while maximizing key information retention. For both pre‐ and post‐mutation sequences, the DNA embeddings and ATAC‐seq data for each segment were averaged pooled, and concatenated.

The concatenated embeddings were then fed into Methven's deep learning module. This module consists of two stacked Bidirectional Gated Recurrent Unit (BiGRU) layers,^[^ ^38^ ^]^ followed by batch normalization layers^[^ ^39^ ^]^ and fully connected layers.^[^ ^40^ ^]^ Methven supports two independent tasks: classification to predict the direction of the SNP's impact on CpG methylation levels (up‐regulation/down‐regulation), and a regression to estimate the magnitude of this impact (slope). By separating these tasks, Methven minimizes task interference and achieves higher predictive accuracy, particularly in determining the direction of the methylation impact.

### 2.2 Methven Achieves High Accuracy in Internal Validation across Genomic Distances

**Figure 2:**
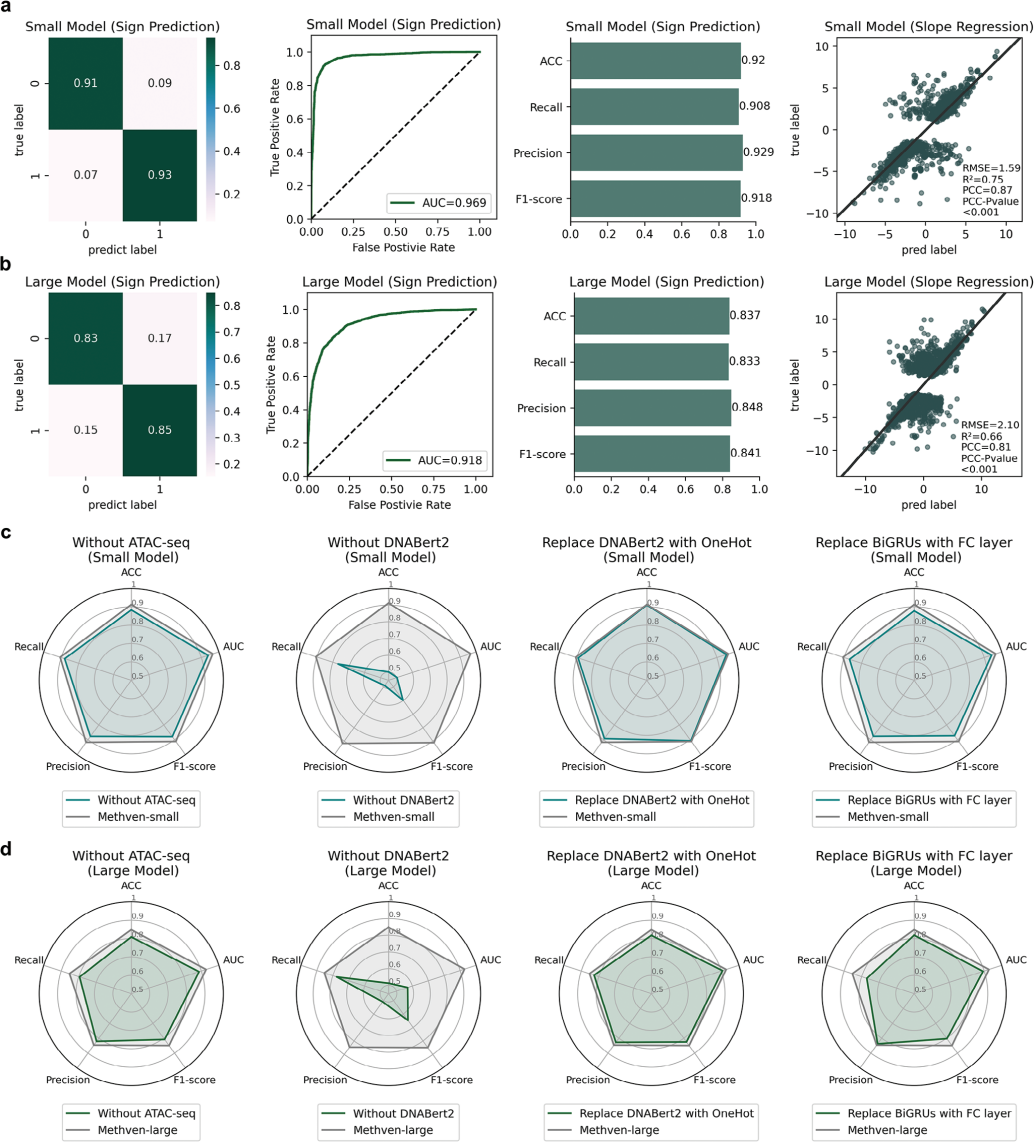
Benchmarking and robustness evaluation of Methven on intra‐dataset. a) Performance of Methven on the small dataset (test samples = 1,988). The classification task is evaluated using a confusion matrix, ROC curve, AUC, accuracy (ACC), recall, precision, and F1‐score. The regression task performance is quantified by RMSE, R^2^, and PCC, with the significance of PCC assessed under the condition of P‐value < 0.001. b) Performance of Methven on the large dataset (test samples = 3,032). Evaluation metrics are applied similarly to those in (a). c) Ablation study on the small dataset. Four ablation experiments were conducted: removing the input ATAC‐seq data, removing the DNA embedding, replacing DNABert2 embedding with OneHot, and replacing the BiGRU layers with fully connected layers. Grey lines represent the performance of the full Methven model, while green lines depict the performance of the ablated models. d) Similar to (c), with the same ablation experiments conducted on the large dataset.

Methven's prediction performance was first evaluated using an internal test set (partitioned from the same dataset as the training set, see “Methods”, **Figure** **2**a,b). Specifically, the dataset used for training and testing comprised 50,190 meQTL data from the meQTL EPIC Database, focusing on CD4+ T cells, alongside corresponding single‐cell ATAC‐seq data from the EpiMap Repository. This dataset was chosen for its comprehensive annotation of SNP‐CpG interactions and high relevance to immune regulation and autoimmune diseases.

The internal test set was generated by splitting the original dataset into training (80%), validation (10%), and testing (10%) sets. Care was taken to ensure that the test set contained SNP‐CpG pairs that represented both small and large SNP‐CpG distances (<10 kbp and 10 kbp‐100 kbp, respectively). The test set included 1,988 small‐distance SNP‐CpG pairs and 3,032 large‐distance SNP‐CpG pairs, enabling Methven's performance to be evaluated across a wide range of genomic distances (Table S1, Supporting Information).

In the classification task, predicting the direction of the SNP's impact on CpG methylation without considering the magnitude helped distinguish directional effects and reduce interference from small absolute slope values. On the small distance SNP‐CpG pairs set, the Methven classification model achieved an ACC of 0.920 and an AUC of 0.969. For the large distance SNP‐CpG pairs set, the ACC was 0.837, and the AUC was 0.918. These results demonstrated the robust prediction capability of the Methven classification model, with a receptive field extending up to 100 kbp.

However, predicting only the direction of the SNP's impact on CpG methylation is insufficient. To address this, additional models were trained to regress the magnitude of the impact, specifically the meQTL slope. The Methven‐small model achieved an RMSE of 1.59 and a Pearson correlation coefficient (PCC) of 0.87 (Student t‐test p < 0.001), while the Methven‐large model recorded an RMSE of 2.10 and a PCC of 0.81 (Student t‐test p < 0.001). The regression task provides finer‐grained predictions and complements the classification task. Together, these two tasks enable Methven to offer both high‐level and detailed insights, improving its overall predictive utility. Furthermore, when the Beta value (meQTL slope) predicted by the regression model has a small absolute value, this indicates that the model considers the mutation to have minimal impact on methylation levels. In such cases, it may be appropriate to classify the mutation as “non‐impactful” and exclude it from annotation as a meQTL.

Ablation experiments revealed that each critical component of Methven independently contributes to its overall performance. These key components include the ATAC‐seq input, DNA embeddings, and the BiGRU layers within the model architecture. We conducted a series of experiments where the ATAC‐seq input was removed, DNA embeddings were excluded, DNABert2 embeddings were replaced with OneHot encoding, and BiGRU layers were substituted with fully connected layers. As shown in Figure 2c,d, the performance degradation following the removal of the essential inputs (both DNA embedding and ATAC‐seq) underscores the validity of the Methven design.

It is worth noting that when using OneHot encoding instead of DNABert2 for generating DNA embeddings, the inability to perform average pooling due to the limitations of OneHot encoding leads to higher computational costs, especially with longer DNA sequences (Tables S3,S4, Supporting Information). Although both embeddings achieved similar performance, DNABert2 exponentially reduced the overall number of model parameters, which is highly beneficial for large‐scale DNA sequence predictions in improving efficiency and scalability.

**Figure 3:**
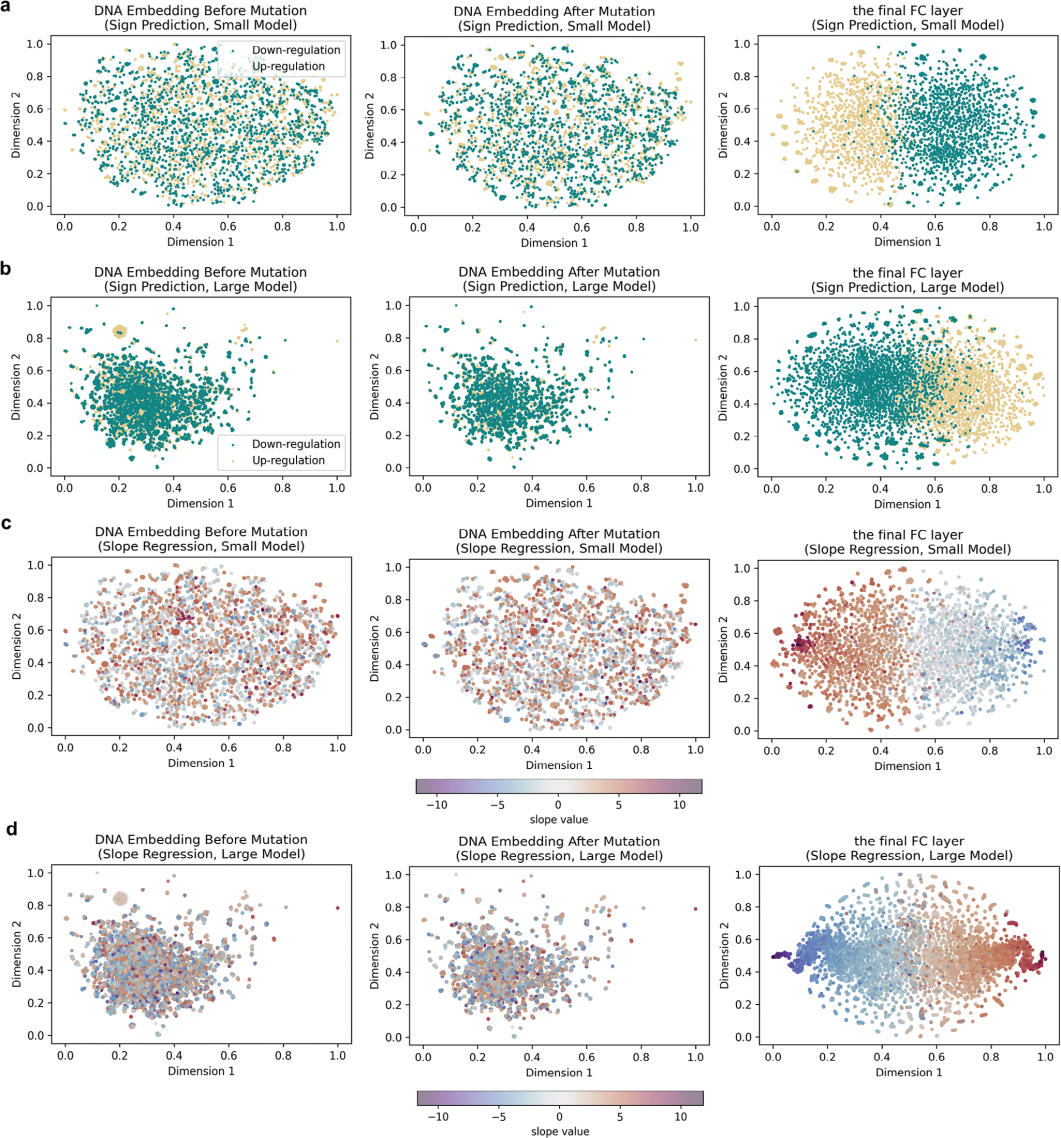
Visualization of representation ability of Methven. a) Visualization of representational ability in the Methven small model for the classification task. t‐SNE was used to perform dimensionality reduction and visualization on the DNA embeddings both pre‐ and post‐mutation, as well as on the outputs from the Methven model after the deep learning module. Green points represent SNP‐CpG pairs where the CpG methylation level increases, while yellow points represent pairs where the CpG methylation level decreases. b) Similar to (a), with t‐SNE applied to the large dataset model. c) Visualization of representational ability in the Methven small model for the regression task. t‐SNE was used to perform dimensionality reduction and visualization on the DNA embeddings both pre‐ and post‐mutation, as well as on the outputs from the Methven model after the deep learning module. Points are colored with a gradient from blue to red, representing slope values from low to high. d) Similar to (c), with t‐SNE applied to the large dataset model.

To further verify the representation learning ability of Methven, we utilized the t‐SNE algorithm^[^ ^41^ ^]^ to visualize the sample distribution based on the initial feature set (pre‐ and post‐mutation DNA embeddings) and the representation generated by Methven. All embeddings and representations were mapped into a two‐dimensional space. To visualize the sample distribution, SNP‐CpG pairs were colored according to the SNP's effect on methylation (**Figure** **3**). We observed that, in both the classification and regression tasks, SNP‐CpG pairs with different labels or slopes were completely intermixed in the two‐dimensional space of the initial characterization. However, in the Methven representation space, SNP‐CpG pairs were separated according to their classification labels (Figure 3a,b) and were distributed in an orderly manner according to slope values in the regression task (Figure 3c,d). These results indicate that Methven is able to efficiently generate high‐quality representation vectors for mutation effect prediction and maintain consistent performance in different tasks.

### 2.3 Methven Outperforms Existing Methods in Predicting Non‐Coding Mutation Effects

While Methven demonstrated high accuracy in internal validation, its true robustness lies in how it compares to other state‐of‐the‐art methods for predicting methylation changes induced by non‐coding mutations.

Methven is capable of predicting the impact of SNPs on all CpG sites within a 100 kbp range, both upstream and downstream, whereas the previous state‐of‐the‐art model, CpGenie,^[^ ^27^ ^]^ was limited to a 500 bp range. To comprehensively demonstrate Methven's effectiveness and robustness in predicting the effects of non‐coding mutations on methylation, we compared it with existing external tools on the classification task. CpGenie, a widely used method specifically designed for predicting the impact of mutations on methylation, served as the primary baseline. To ensure a fair comparison, we applied CpGenie to data with SNP‐CpG distances up to 100 kbp, consistent with the range used in Methven.

We also included Enformer^[^ ^42^ ^]^ in our comparisons. While Enformer was originally designed to predict the impact of non‐coding mutations on gene expression, it excels at generating functional annotations of DNA sequences. On the other hand, Methven's features are derived from large‐scale pretraining on DNA sequences to learn semantic representations that capture deeper relationships within the genome. Comparing Methven with Enformer allowed us to evaluate which approach—functional annotations or semantic information—offers stronger predictive capabilities in assessing the impact of non‐coding mutations on methylation.

**Figure 4:**
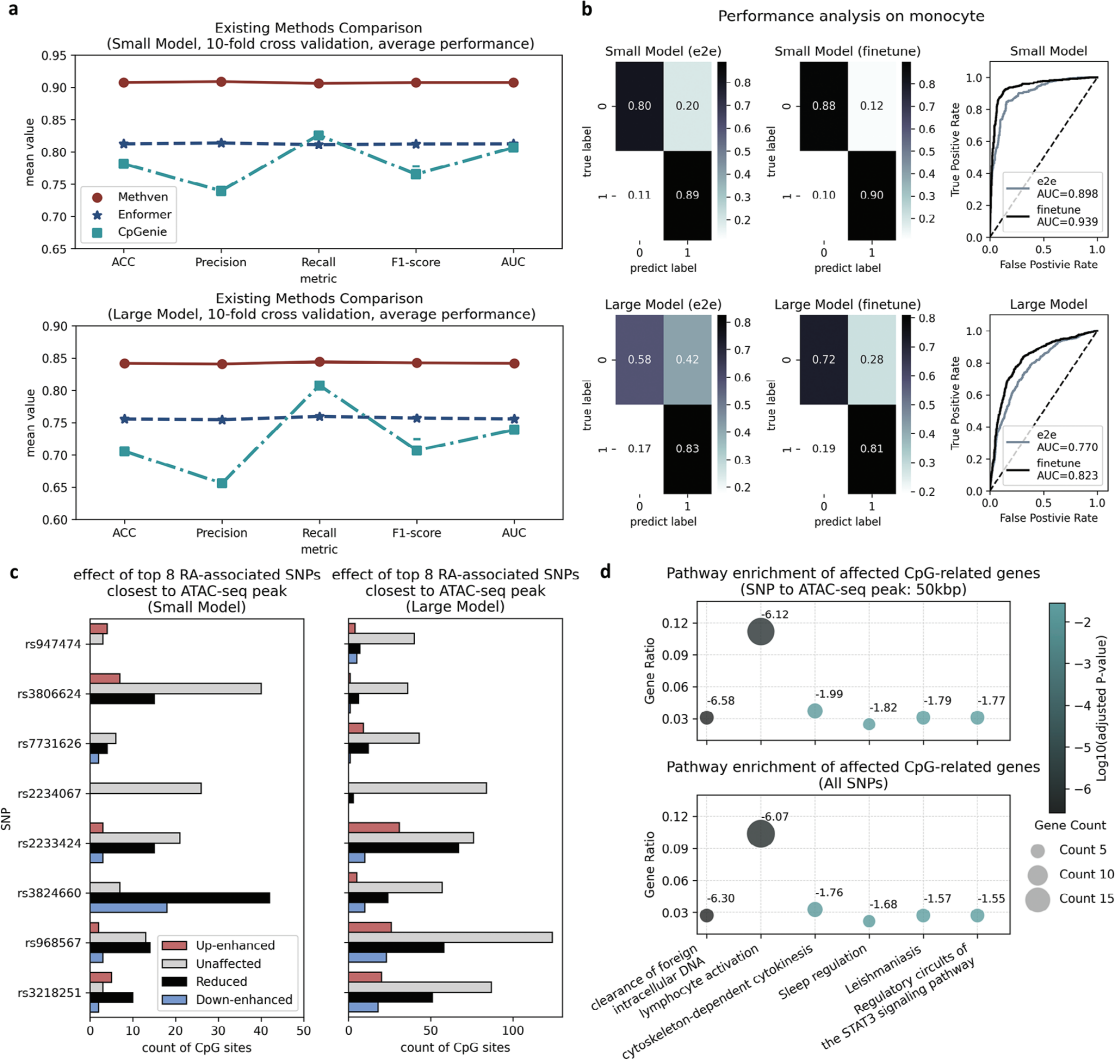
External validation of Methven on existing methods, new cell type, and disease‐associated SNPs. a) Comparison of ten‐fold cross‐validation performance between Methven, Enformer, and CpGenie on the classification task (Table S5, Supporting Information). The metrics used for comparison include ACC, Precision, Recall, F1‐score, and AUC. To ensure a fair comparison of these models' ability to learn the relationship between SNPs and CpG sites, embeddings from the layer preceding the output layer of each model were extracted, and a decision tree with identical parameters was trained on these embeddings. b) Methven's performance on monocyte single‐cell meQTL datasets. The experiments involved two approaches: end‐to‐end (e2e) training of Methven directly on the monocyte dataset, and fine‐tuning Methven pre‐trained on the CD4+ T cell dataset. c) Analysis of rheumatoid arthritis (RA)‐associated SNPs using the Methven regression model. The SNPs predicted were selected from genome‐wide association studies (GWAS) analysis as RA‐associated SNPs. Red bars indicate SNPs where the absolute difference in slope between case and control SNPs is greater than 0.5, with the control SNP having a positive slope, suggesting an up‐enhancement of methylation impact in RA cases. Blue bars represent SNPs where the absolute difference in slope is greater than 0.5, with the control SNP having a negative slope, indicating a down‐enhancement of methylation impact in RA cases. Grey bars indicate SNPs where the absolute difference in slope is less than 0.5, suggesting little association with RA in terms of methylation impact. Black bars denote SNPs where the absolute slope in RA cases is smaller than in controls, indicating a reduced impact on methylation in RA conditions. d) Pathway enrichment results for genes corresponding to Methven‐predicted affected CpGs. To compare the impact of varying signal‐to‐noise ratios, the SNPs within a 50 kbp range of the ATAC‐seq peak were analyzed separately and compared to the results from all SNPs.

The comparison was conducted using ten‐fold cross‐validation on the classification task. To fairly assess the representational power of different methods, we extracted the embeddings from the penultimate layer of each model (the embeddings used for final classification, representing the highest‐level features learned by the model) and then trained a decision tree with default parameters on these embeddings, evaluating performance on an internal test set (**Figure** **4**a; Figure S3, Supporting Information).

Methven outperformed all other methods across both small and large datasets on all evaluation metrics, consistently demonstrating balanced recognition of both positive and negative samples. Specifically, the Methven‐small model achieved a mean of ACC = 0.908 and a mean of AUC = 0.908, while the Methven‐large model recorded a mean of ACC = 0.842 and a mean of AUC = 0.842 (Table S5, Supporting Information). In contrast, while Enformer also demonstrated balanced recognition of positive and negative samples, its overall performance was lower than Methven's, likely because the functional annotation features learned during the pretraining of Enformer are not fully optimized for tasks focused on methylation tasks.

CpGenie, which relies on OneHot encoding for DNA sequences and convolutional neural networks (CNN) for representation learning, struggled with longer sequence lengths (>500 bp reported), resulting in less stable and poorer performance CpGenie achieved a mean of ACC = 0.782 and a mean AUC = 0.807 for small‐distance pairs, and a mean ACC = 0.706 and a mean AUC = 0.739 for large‐distance pairs (Table S5, Supporting Information). These findings demonstrate that Methven effectively predicts the impact of non‐coding mutations on DNA methylation and offers improved generalization across genomic distances compared to state‐of‐the‐art methods.

It is worth highlighting that even if CpGenie and Enformer were extended to single‐cell resolution, their assessment of SNP effects within specific cell types would remain statical, as both rely exclusively on DNA sequence information for predictions. In contrast, Methven offers a unique advantage by incorporating personalized ATAC‐seq data as input, introducing greater flexibility. This enables Methven to identify distinct patterns for the same cell type across different individuals or to evaluate SNP effects across various stages of a disease—an adaptability not achievable with previous methods.

### 2.4 Methven Effectively Generalizes to Monocytes for Methylation Prediction

In addition to its strong performance compared to existing methods, we also sought to explore Methven's generalizability across different cell types, starting with monocytes. Mutations can have different effects depending on the cellular environment. To assess Methven's ability to generalize to other cell types, we applied the Methven classification model to a dataset comprising monocyte single‐cell meQTLs. This dataset was downloaded from the EPIGEN MeQTL Database,^[^ ^35^ ^]^ with corresponding ATAC‐seq data sourced from the EpiMap Repository.^[^ ^36^ ^]^ After preprocessing, the number of SNP‐CpG pairs used for training and testing was approximately one‐third of those in Methven's internal dataset (Tables S1,S8, Supporting Information).

We initially trained Methven directly on the monocyte external validation dataset using an end‐to‐end (e2e) approach. We observed solid classification performance on both the small SNP‐CpG pairs and large SNP‐CpG pairs (AUCs of 0.898 and 0.770, respectively, Figure 4b, Table S6, Supporting Information), indicating its potential to generalize to other cell types or tissue types, provided that corresponding ATAC‐seq data is available.

Next, we fine‐tuned Methven based on the pre‐trained model from CD4+ T cells. This fine‐tuned model showed a slight improvement in performance over the end‐to‐end training, with AUCs of 0.939 and 0.823, respectively (Figure 4b). This improvement is likely due to the larger training data size in Methven's internal dataset, which helped capture intrinsic relationships between meQTLs and ATAC‐seq. These results suggest that Methven could serve as a generalized pre‐trained model, especially as the amount and the cell/tissue type of training data increase over time.

### 2.5 Methven Reveals Methylation Changes Linked to Disease‐Associated SNPs in Rheumatoid Arthritis

After validating Methven's ability to generalize to different cell types, we applied it to uncover potential links between SNP‐induced methylation changes and specific diseases, focusing on rheumatoid arthritis (RA). In real‐world studies, investigating the effects of mutations often involves exploring disease mechanisms.^[^ ^43^, ^44^, ^45^ ^]^ Methven's integration of ATAC‐seq inputs enables the analysis of mutations occurring in the same cell type under different disease processes. To evaluate Methven's potential in uncovering mutation‐disease connections, we selected SNPs highly associated with RA through genome‐wide association studies (GWAS) analysis.^[^ ^46^ ^]^ For training, we used ATAC‐seq data from CD4+ T cell lines, with cells stimulated for 24 hours with anti‐CD3/CD28 serving as the case ATAC‐seq, and unstimulated cells as the control ATAC‐seq.^[^ ^47^ ^]^ To enhance the signal‐to‐noise ratio, we filtered SNPs located within 1 kbp upstream and downstream of the control ATAC‐seq peak regions since these regions are highly enriched for regulatory elements,^[^ ^48^ ^]^ resulting in 8 remaining SNPs. All CpG sites within a 100 kbp range upstream and downstream of these SNPs were annotated.

We then mapped the predicted affected CpGs to the nearest transcription start site (TSS). To enhance the signal‐to‐noise ratio, we conducted pathway enrichment analysis for the affected CpG‐related genes observed by all SNPs and the SNPs located within a 50 kbp range of the ATAC‐seq peaks. We then identified six pathways, such as “clearance of foreign intracellular DNA” and “lymphocyte activation”, with significantly adjusted P‐values (Mann‐Whitney U test P‐value < 0.05) that were common to both groups (Figure 4d). The pathway of clearance of foreign intracellular DNA is reported closely linked to the pathogenesis of RA through its role in the immune response and inflammation,^[^ ^49^ ^]^ while the signaling lymphocytic activation molecule family (SLAMF) may influence RA pathogenesis by participating in inflammation mediated by infiltrating immune cells.^[^ ^50^ ^]^ The identification of RA‐associated CpGs influenced by SNPs and recognized by Methven, which align with pathways known to be involved in RA pathogenesis, demonstrates Methven's capability to enhance the understanding of the role of “mutations affect methylation levels” in disease mechanisms.

Next, we applied Methven's regression model to predict the impact of these SNPs in both case and control samples with corresponding ATAC‐seq data. Based on the differences in slopes between the two conditions, we categorized the annotated CpG sites into four groups: those where mutation impact on methylation was enhanced by disease occurrence (Up‐enhanced), negatively enhanced (Down‐enhanced), reduced impact (Reduced), or unaffected by the disease (Unaffected). Methven was able to distinguish among these four categories of CpG sites (Figure 4c), addressing a gap left by other methods in this area.

**Figure 5:**
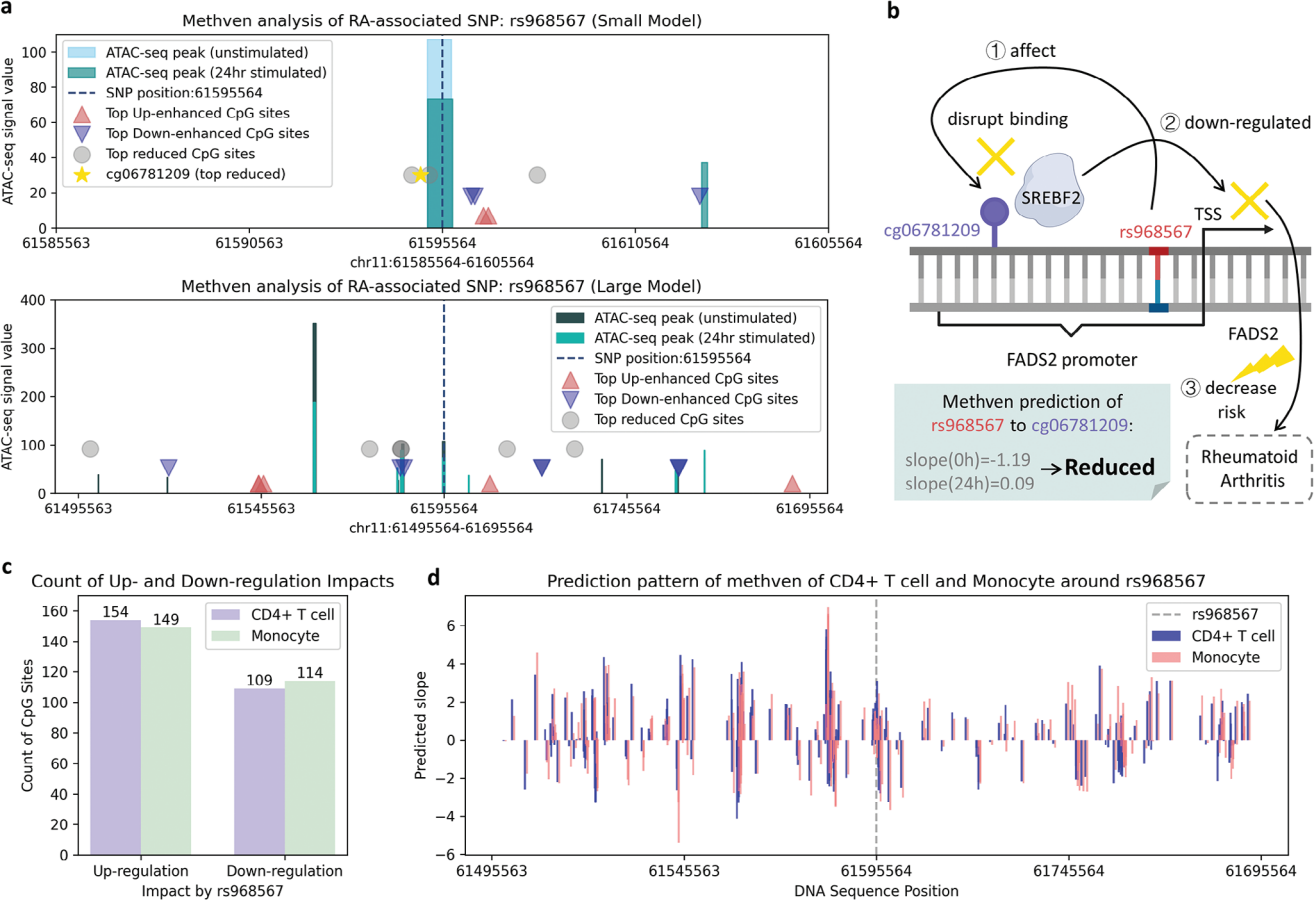
Methven analysis of rs968567 on disease process, a case of rs968567‐CpG associated pair, and pattern analysis across different cell types. a) Visualization of RA‐associated SNP analysis for the Methven. rs968567 has been reported as a key SNP for RA. For the different categories of SNPs identified in Figure 4(c), top‐ranking CpG sites were selected as Top CpG sites, showing that most are located near ATAC‐seq peaks. b) Case study of Methven's prediction for the rs968567 to cg06781209 interaction. cg06781209 is a binding site for the transcription factor SREBF2. rs968567 in the promoter region of the FADS2 gene alters DNA methylation, disrupting the binding of SREBF2 and downregulating FADS2 expression, thereby reducing RA risk. In Methven's predictions, the absolute value of the unstimulated slope is greater than that of the case slope, indicating that in RA, the CpG site is less influenced by the SNP. This suggests a reduced ability of the SNP to modulate CpG methylation, thereby failing to suppress RA as effectively. c) Bar plot of the count of up‐regulated and down‐regulated CpG sites affected by rs968567 in CD4+ T cells and monocytes. CD4+ T cells have slightly more up‐regulated sites (154) compared to monocytes (149), while down‐regulated sites are nearly equal between the two cell types. This indicates rs968567's similar but distinct regulatory impacts on CD4+ T cells and monocytes. d) Line plot of the predicted regulatory slope of Methven for CD4+ T cells and monocytes across the CpG site around rs968567. The patterns for both cell types are largely consistent, though subtle differences in slope magnitude reflect slight variations in the regulatory effect of rs968567 between the two cell types.

As an example, we examined rs968567, an SNP proven to be highly associated with RA,^[^ ^51^ ^]^ and analyzed its impact on case and control ATAC‐seq peaks, along with the distribution of CpG sites across the four categories relative to the SNP (**Figure** **5**a, Table S7, Supporting Information). We observed that the CpG sites with high predicted scores of each category (top CpG sites) were clustered around the ATAC‐seq peak regions, which likely contain functional regulatory elements. This clustering may explain why these CpG sites were more affected by the SNP and exhibited greater changes due to disease occurrence.

One CpG site, cg06781209, is a binding site for the transcription factor SREBF2. The SNP rs968567, located in the promoter region of the *FADS2* gene, has been reported to alter the methylation level of cg06781209.^[^ ^52^, ^53^ ^]^ This alteration disrupts the binding of SREBF2, downregulating *FADS2* gene expression and subsequently reducing RA risk.^[^ ^52^, ^53^ ^]^ In other words, changes in the methylation level of cg06781209 are associated with the suppression of the effects of rs968567, thereby influencing RA risk. Methven's predictions showed that the SNP's influence on CpG methylation was less pronounced in the 24‐hour stimulated condition than in the unstimulated condition (Figure 5b, 24‐hour stimulated predicted slope = 0.09, unstimulated predicted slope = ‐1.19), suggesting diminished transcription factor binding and consequently reduced RA risk. These findings align with previous studies showing that the methylation of cg06781209 can suppress the effects of rs968567, thus influencing RA risk.^[^ ^52^, ^53^ ^]^

These results demonstrate that Methven can assist in determining whether the pathogenicity of disease‐associated SNPs is mediated by “changes in methylation affected by SNPs” thus providing insights into the impact of individual mutations on disease risk and progression, and supporting the development of personalized treatment strategies.

### 2.6 Methven Captures Cell Type‐Specific Regulatory Patterns of SNP‐Induced Methylation

Given the importance of cell type‐specific methylation patterns in understanding disease risk, we next evaluated Methven's ability to capture such regulatory patterns across different cell types, specifically CD4+ T cells and monocytes. We applied Methven to assess the regulatory effects of SNP rs968567 on CpG sites in both cell types, focusing on up‐ and down‐regulation patterns (Figure 5c), as well as the predicted regulatory slopes (Figure 5d). The analysis revealed a balanced distribution of up‐ and down‐regulated CpG sites between the two cell types. CD4+ T cells showed slightly more up‐regulated sites (154) than monocytes (149), while the number of down‐regulated sites was comparable (109 in CD4+ T cells and 114 in monocytes). This balance demonstrates Methven's ability to capture cell type‐specific regulatory responses even when the underlying genetic perturbation, such as the SNP rs968567, is the same.

When a disease‐associated SNP like rs968567 exerts specific methylation impacts on particular cell types, Methven can assess the consistency of methylation patterns across cell types. By comparing the predicted methylation changes with known patterns, clinicians may stratify individuals into high‐ or low‐risk categories based on their methylation response. This underscores Methven's potential in precision medicine, where cell type‐specific epigenetic changes can help predict individual disease susceptibility.^[^ ^54^ ^]^

### 2.7 Leverages Functional DNA Regions for Enhanced Methylation Predictions

To further illustrate Methven's prediction capabilities, we investigated its ability to leverage functional DNA regions, which play a crucial role in regulating gene expression and methylation. Non‐coding DNA often contains regulatory elements that influence both gene expression and methylation patterns. SNPs located near these functional regions are more likely to have an impact on these processes. To explore Methven's ability to understand and utilize sequence features, we aligned the hidden states of the BiGRU layers with the DNA sequences and analyzed them based on different functional regions, as well as whether the DNA bases were located within these functional regions. We obtained annotations for eight types of functional regions from the UCSC Genome Browser: active promoter, strong enhancer, transcriptional transition, transcriptional elongation, insulator, heterochrome, repressed region, and repetitive element/copy number variation.

**Figure 6:**
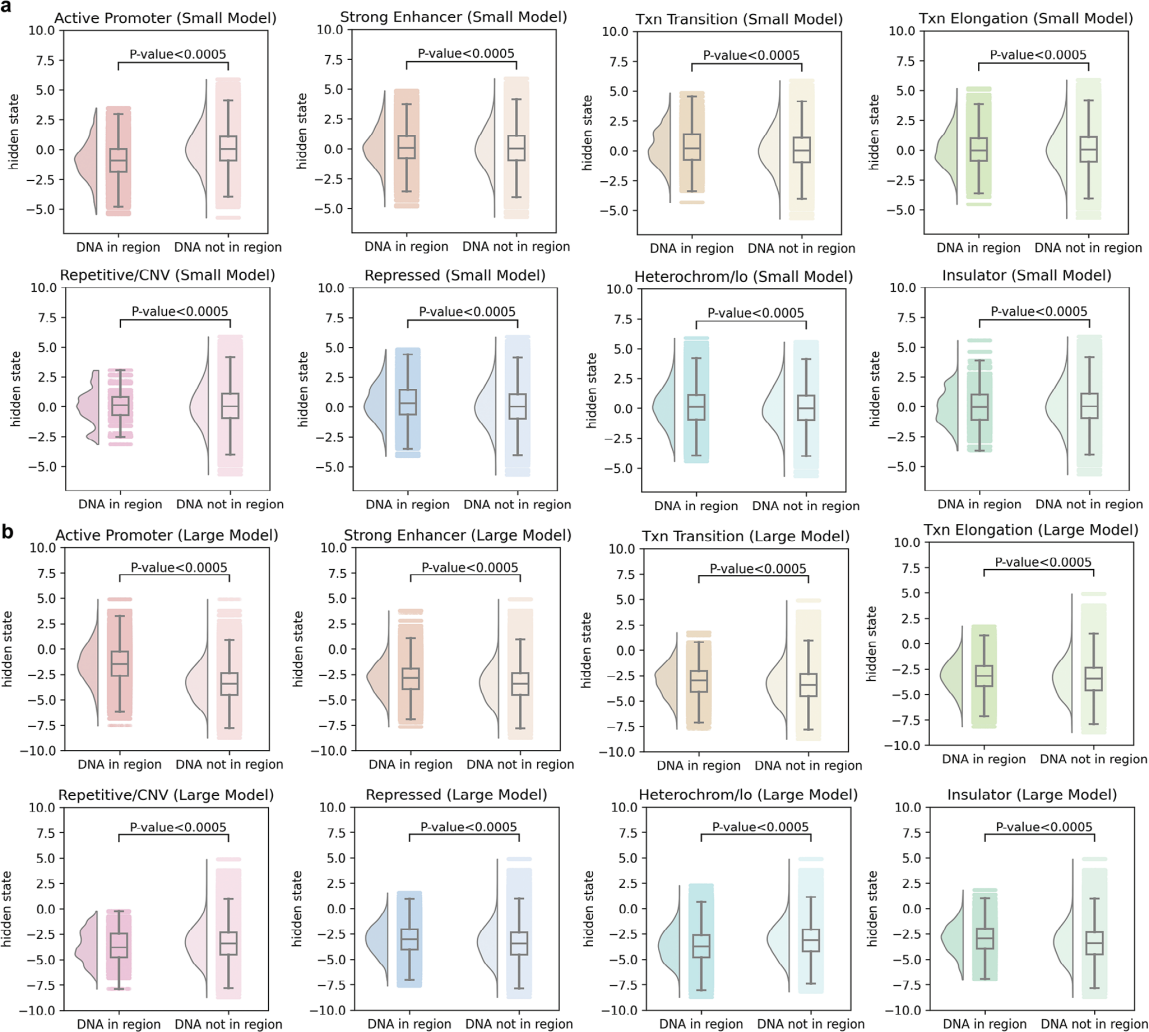
Hidden state analysis of Methven. a) Differences in the hidden states of stacked BiGRU layers in the Methven small model between functional and non‐functional regions. Eight functional regions were annotated using the UCSC Genome Browser and mapped onto the DNA sequences of the input SNP‐CpG pairs (Txn Transition: transcriptional transition, Txn Elongation: transcriptional elongation, Repetitive/CNV: repetitive element/copy number variation, Repressed: repressed region, Heterochrom/lo: heterochrome). The differences between functional and non‐functional regions were assessed using the Mann‐Whitney U test. b) Similar to (a), with the analysis conducted using the Methven large model.

The hidden states of the BiGRU layer showed significantly different activation values (Mann‐Whitney U test, P‐vlaue < 0.005) between functional regulatory regions and non‐functional regions (**Figure** **6**), suggesting that Methven can recognize and incorporate information from critical genomic regions. This ability to differentiate functional from non‐functional regions likely contributes to Methven's high classification performance in predicting the effects of non‐coding mutations on DNA methylation.

Additionally, we observed differences in the distribution of hidden state activations between the Methven‐small and Methven‐large models. These differences suggest that Methven learns distinct regulatory patterns for short‐range and long‐range interactions. This observation reinforces the decision to train separate models for different genomic distances, as it highlights Methven's capacity to adapt to the unique regulatory mechanisms that operate at various scales within the genome.

## 3 Discussion

Accurately predicting the epigenetic consequences of non‐coding mutations on DNA methylation, particularly at single‐cell resolution, remains a significant challenge in understanding gene regulation and its links to complex diseases. Despite advances in GWAS that have identified numerous genetic variants associated with diseases and traits, pinpointing causal variants and clarifying their pathogenic mechanisms remain difficult. Previous tools, such as CpGenie^[^ ^27^ ^]^ have pioneered non‐coding variant effect prediction on DNA methylation, but their limited receptive fields and static prediction capabilities hinder their application in broader genomic and cellular contexts. Similarly, models like DeepSea^[^ ^29^ ^]^ and Enformer^[^ ^42^ ^]^ provide valuable functional annotations from DNA sequences but struggle to account for the dynamic and cell‐specific regulatory changes that are critical for understanding disease progression.

Methven addresses these limitations by integrating DNA sequences with single‐cell ATAC‐seq data, modeling SNP‐CpG interactions over genomic distances up to 100 kbp using a divide‐and‐conquer strategy. This approach allows Methven to capture both short‐ and long‐range regulatory interactions with greater accuracy than previous methods. Moreover, Methven's architecture supports predictions at single‐cell resolution, moving beyond static predictions to model the dynamic interactions between non‐coding mutations and the epigenome. By leveraging DNABert2 embeddings and single‐cell ATAC‐seq data, Methven predicts both the direction and magnitude of methylation changes, achieving a classification accuracy of 92.0% and an AUC of 0.969 for short‐range interactions. These improvements highlight Methven's flexibility in modeling complex genomic interactions, crucial for advancing our understanding of epigenetic regulation. By utilizing single‐cell ATAC‐seq data, Methven can dynamically learn the relationship between DNA sequences and the chromatin accessibility of the specific sample being predicted, enabling personalized predictions that would not be achievable with the prediction based only on DNA sequences.

Methven introduces several advancements in the field of computational genomics. First, its divide‐and‐conquer strategy effectively captures both local and long‐range SNP‐CpG interactions, a feature that distinguishes Methven from earlier tools limited to short‐range predictions. Second, it utilizes DNABert2 to generate pre‐trained DNA embeddings, efficiently encoding complex regulatory relationships while maintaining a lightweight architecture for large‐scale predictions. Third, Methven is the first tool to predict non‐coding mutation effects on DNA methylation at single‐cell resolution, making it an important innovation for studying cell‐type‐specific regulation in diseases like cancer and autoimmune disorders. Additionally, the dual‐task architecture, supporting both classification and regression outputs, enables Methven to predict not only the direction but also the magnitude of methylation changes, contributing to a more comprehensive understanding of epigenetic regulation.

The Results demonstrate that Methven's predictive power is validated through its strong performance on the internal CD4+ T cell dataset, as well as its ability to generalize to an external monocyte dataset. Methven showed robust cell‐type‐specific predictions, particularly in identifying methylation changes associated with disease‐related SNPs, such as those linked to rheumatoid arthritis. The fine‐tuning of Methven on monocytes, originally trained on CD4+ T cells, further highlights its adaptability across different biological contexts, an important advantage over prior tools that often struggle to generalize beyond their training datasets.

Methven's application to RA‐associated SNPs uncovered significant mechanistic links between non‐coding mutations and disease pathogenesis. By integrating ATAC‐seq data, Methven successfully captured both the spatial and temporal dynamics of chromatin accessibility, a critical factor in understanding how regulatory elements evolve over time in diseases like RA. Notably, Methven identified CpG sites involved in pathways such as “clearance of foreign intracellular DNA” and “lymphocyte activation”, both of which are pivotal to RA pathogenesis. This capability of Methven to integrate epigenetic data and predict cell‐type‐specific methylation responses provides significant potential for precision medicine, where it could be used to stratify patients based on their epigenetic risk profiles.

In addition to predicting which CpG sites are affected by disease‐associated mutations, Methven is also able to categorize these sites based on the extent of methylation changes (e.g., up‐regulated, down‐regulated, reduced, or unaffected). This granularity in prediction offers a detailed understanding of how non‐coding variants affect gene expression through methylation changes, which is crucial for diseases driven by immune dysregulation, such as RA. For instance, Methven accurately predicted the impact of rs968567, an SNP strongly associated with RA, on the methylation of cg06781209, a CpG site involved in regulating the expression of *FADS2*. The model's predictions aligned with prior findings showing that changes in methylation at this site contribute to disease risk, demonstrating the utility of Methven in identifying actionable epigenetic biomarkers.

Despite its strengths, Methven has limitations. One key issue is the limited availability of high‐quality single‐cell meQTL datasets obtained through fine‐mapping, which has restricted the current version of Methven. To address this limitation, we plan to develop computational approaches capable of generating large‐scale single‐cell meQTL datasets across diverse cell types, thereby enhancing Methven's pre‐training process. As an exploratory analysis, we conducted a preliminary evaluation of Methven's generalizability to tissue‐level meQTLs using a small‐scale retina meQTL dataset (Figure S4, Supporting Information). Importantly, Methven's primary contribution lies in providing a pattern for learning the regulatory relationships between DNA sequences and epigenetic information. Therefore, Methven has the potential to be extended to other downstream tasks related to mutation impact prediction (Figure S5, Supporting Information).

Another issue worth discussing is Methven's reliance on high‐quality cell‐specific ATAC‐seq data. These data capture cell‐type‐specific chromatin states, which were shown to be beneficial to Methven's performance through ablation experiments. While recent advances in sequencing technologies are gradually increasing the availability of such datasets, their high cost and technical requirements may still pose challenges for wider adoption. To overcome this, future work will explore the integration of complementary omics data, such as histone modification patterns and chromatin interaction maps, to expand Methven's applicability and further refine its predictions.

Future research should also refine Methven's pretraining strategies. As Methven serves as a theoretical framework, embeddings from other language models can be used in place of OneHot encoding for sequence representation. With the ongoing development of new DNA pretraining models, we will continue to track advancements in this field and test additional models to enhance Methven's performance and applicability. Additionally, while Methven currently operates within a 100 kbp receptive field, future research will focus on exploring strategies to extend its range to even larger regulatory domains. We will also work on balancing the computational cost and ensuring model accuracy for these extended ranges. In summary, Methven's dynamic, cell‐specific approach offers insights into the epigenetic impact of non‐coding mutations and holds promise for both basic research and personalized medicine.

## 4 Experimental Section

### Datasets

***The meQTL EPIC Database***: The meQTL EPIC dataset^[^ ^35^ ^]^ was downloaded from the meQTL EPIC Database website (https://epicmeqtl.kcl.ac.uk/), which reported the results of a meQTL analysis at 724,499 CpGs profiles in 2,358 blood samples from three UK cohorts. In this study, meQTL data from CD4+ T cells were obtained in the EPIC meQTL Database, which as the intra‐dataset was used. Additionally, monocyte meQTL data was utilized from the same database as one of the external validation datasets.

***EpiMap Repository***: The corresponding ATAC‐seq data for matching CD4+ T cell meQTL and monocyte meQTL were downloaded from the EpiMap Repository^[^ ^36^ ^]^ (https://compbio.mit.edu/epimap/), which includes aggregated and uniformly re‐processed functional genomics data from 3030 references across sources such as ENCODE and Roadmap.

***GWAS Summary of RA risk SNPs***: The summary results of RA risk SNPs in 101 risk loci were derived from a three‐stage trans‐ethnic meta‐analysis, involving a genome‐wide association study (GWAS) of over 100,000 subjects of European and Asian ancestries.^[^ ^46^ ^]^

***RA‐Stimulated and Unstimulated ATAC‐seq of CD4+ T Cells***: In the disease‐SNP analysis, the ATAC‐seq data generated from CD4+ T cells were downloaded at different time points (0 min, and 24 h) after stimulation with anti‐CD3/anti‐CD28 which contains information about chromatin dynamics associated with rheumatoid arthritis (RA).^[^ ^47^ ^]^

### Data Pre‐Processing

***Balanced Sampling***:For each SNP, CpG sites within a 100 kbp range upstream and downstream that exhibit methylation level changes were identified. SNP‐CpG pairs with distances less than 10 kbp formed the small dataset, while those with distances between 10 kbp and 100 kbp comprised the large dataset. To prevent model bias, the dataset was balanced by down‐sampling the more dominant classification category according to chromosome distribution, ensuring that the number of positive and negative samples was approximately equal (Table S1, Supporting Information). This balanced dataset was then used as the intra‐dataset for both the classification and regression tasks.

**Fetch Initial Input Feature**: For each SNP‐CpG pair, DNA sequences and corresponding ATAC‐seq data were extracted with the CpG site at the center, spanning a radius of 10 kbp or 100 kbp, ensuring the SNP falls within this range. The sequences, both pre‐ and post‐mutation, were subjected to positional‐wise cutting centered on the CpG site, and the resulting fragments were input into the DNA language model to generate DNA embeddings.

***Embedding Generation***: Due to the maximum input length constraint of DNABert2, the DNA sequences must be segmented. By centering each cut on the CpG site—an essential point of interest—this segmentation ensures that each fragment remains within the permissible input length of the DNA language model while maximizing the retention of critical information integrity.

The positional‐wise cutting algorithm can be described as follows: given a DNA sequence/ATAC‐seq centered on a CpG site (with a total length of 10,001 bp for small pairs and 100,001 bp for large pairs), the first and last cuts were 250 bp in length, while a fragment centered on the CpG site, with a length of 501 bp, forms the central cut. The remaining DNA sequence between the terminal cuts and the central cut was uniformly segmented into cuts of 500 bp each. Specifically, the DNA sequence between the first cut and the central cut was divided into n cuts of 500 bp, with the same applied to the sequence between the central cut and the last cut. For the small model, n equals 19, and for the large model, n equals 199 (Figure 1b).

Finally, the DNA embeddings from each cut and the ATAC‐seq data underwent average pooling and were concatenated.

### Benchmarking

***Definition of Classification and Regression Task***: In this study, two independent tasks were designed: a classification task to predict the direction of the SNP's impact on CpG methylation levels (up‐regulation/down‐regulation) and a regression task to predict the magnitude of this impact (slope). In the classification task, samples with a slope greater than 0 were defined as positive samples (up‐regulation), while those with a slope less than 0 were defined as negative samples (down‐regulation). This layered prediction approach allows Methven to focus on each predictive goal independently, minimizing task interference and achieving higher predictive accuracy, particularly in determining the direction of the methylation impact.

***Evaluation Metrics***: For the classification task, Accuracy (ACC), Precision, Recall, F1‐score, and Area Under the Curve (AUC) were employed to assess model performance. For the regression task, Root Mean Square Error (RMSE), R‐squared, and Pearson Correlation Coefficient (PCC) were utilized as key metrics.

***Dataset Split***: In this study, the intra‐dataset was divided using two distinct strategies. The first strategy was designed to facilitate model performance comparisons and was structured to support ten‐fold cross‐validation. The second strategy was applied for finalizing and releasing the model, involving a random split of the data into training, validation, and testing sets in an 8:1:1 ratio.

### Training Details of Methven

Methven was built and trained using the TensorFlow 2.7 library on an NVIDIA 3,090 GPU (Table S2, Supporting Information). For the classification task, the model was trained using binary cross‐entropy as the loss function, while mean squared error (MSE) was employed for the regression task. All models were trained with a learning rate of 0.001, utilizing the Adam optimizer, and an early stopping strategy with patience of 10 epochs was applied.

### Comparison Methods

***CpGenie***: To compare Methven with the current state‐of‐the‐art tool for predicting the impact of non‐coding mutations on methylation, CpGenie was implemented following the official guidelines (https://github.com/gifford‐lab/CpGenie). Since the official version of CpGenie only supports predictions for CpG sites within a 500 bp range upstream and downstream of SNPs, the CpGenie model was extended by expanding the input dimensions while maintaining the identical convolutional layers and hyperparameters (such as the number of filters, kernel size, and stride) as in the original version. The extended CpGenie was trained using the ten‐fold cross‐validation dataset, and an early stopping strategy with patience of 10 epochs was applied (same as Methven). The output was extracted from the layer preceding the final output layer for each fold, representing the highest‐level features learned by the CpGenie model. The extracted representations from each fold were then fed into a decision tree implemented using the scikit‐learn library, with default parameters, to evaluate the performance. For a fair comparison of representation learning capabilities, the same process was applied to Methven.

***Enformer***: Enformer was capable of providing functional annotations for DNA sequences within approximately a 10 kbp range upstream and downstream of SNPs. These annotations were obtained using the official running interface (https://github.com/google‐deepmind/deepmind‐research/tree/master/enformer) and applied to the ten‐fold cross‐validation dataset. For each fold, the extracted functional annotations were also fed into a decision tree implemented with default parameters using the scikit‐learn library (same as Methven and CpGenie). By comparing Methven with Enformer, we aim to evaluate how the representations learned by Methven perform relative to functional annotations in predicting the impact of non‐coding mutations on methylation.

### External Validation on Monocyte Dataset

The processing of monocyte meQTL data followed the same procedure as that used for the intra‐dataset. During end‐to‐end training, the Methven model architecture employed was identical to that used for training on CD4+ T cells, with all model weights initialized randomly. In the fine‐tuning process, however, the initial weights of the monocyte model were derived from the Methven model trained on CD4+ T cells. Regardless of whether end‐to‐end training or fine‐tuning was applied, the training details for the monocyte model were consistent with those used for the CD4+ T cell model.

### Joint Analysis with Disease‐Associated SNPs

***Definition of Affected and Unaffected CpG Sites***: The Methven regression model was used to predict the impact of both the case and control SNPs along with their corresponding ATAC‐seq data. Subsequently, based on the differences in the predicted slopes (i.e., the predicted slope for the case minus the predicted slope for the control), all annotated CpG sites were categorized into four groups: Up‐enhanced (where methylation impact was positively enhanced by disease occurrence), Down‐enhanced (where methylation impact was negatively enhanced), Reduced (where methylation impact decreases), and Unaffected (where methylation impact remains unchanged by disease occurrence). The specific calculation can be defined by the following formula:

(1) $$\begin{array}{c} Affect\;change=\left\{\begin{array}{c} Up-enhanced,\;\left|\Delta slope\right|>0.5\;and\;slope\left(control\right)\geq 0\\ Down-enhanced,\;\left|\Delta slope\right|>0.5\;and\;slope\left(control\right)<0\\ Reduced,\left|\Delta slope\right|>0.5\;and\;\left|slope\left(case\right)\right|<\left|slope\left(control\right)\right|\;\\ Unaffected,\left|\Delta slope\right|\leq 0.5\; \end{array}\right. \end{array}$$

***ATAC‐Seq Data Pre‐processing***: ATAC‐seq data from unstimulated (0h) and 24‐hour stimulated samples were used as control and case ATAC‐seq, respectively. The downloaded ATAC‐seq data were converted to bigwig files using BEDTools^[^ ^55^ ^]^ and BedGraphToBigWig,^[^ ^56^ ^]^ and subsequently aligned to the hg19 genome using CrossMap.^[^ ^57^ ^]^ These data were matched with DNA sequences corresponding to RA risk SNPs and then input into Methven for prediction.

***GO Enrichment and Analysis***: We calculated the distance from each CpG site to its nearest gene and annotated the distance to the gene's TSS. CpG sites were then filtered based on an absolute difference in predicted slopes of 0.5 or greater, indicating significant changes in methylation levels. CpG sites were further focused on located within 2 kb of the TSS,^[^ ^58^ ^]^ as these sites were in the proximal promoter region and may directly influence transcription initiation and gene expression regulation. The filtered CpG sites were then used to identify neighboring genes for pathway enrichment analysis using Metascape.^[^ ^59^ ^]^

## Conflict of Interest

The authors declare no conflict of interest.

## Author Contributions

Z.L. designed the study, completed the model construction, performed the analysis experiments, and wrote the paper. A.G. performed the GO analysis. Y.B. contributed to dataset curation. G.N.L. supervised the work and revised the paper. All authors read and approved the final version of the manuscript.

## Supporting information



Supporting Information

## Data Availability

The data that support the findings of this study are openly available in GitHub at https://github.com/Liuzhe30/Methven.
