# Supplementary material for: Epigenetic Impacts of Non‐Coding Mutations Deciphered Through Pre‐Trained DNA Language Model at Single‐Cell Resolution
Source: Adv Sci (Weinh). 2025 Jan 30;12(11):2413571. doi: 10.1002/advs.202413571 (PMC11924033; doi:10.1002/advs.202413571)

**Supplementary Information**

# Tables

Table S1. The data distribution of the Methven intra-data.

| **Model size** | **type** | **No. of Up-regulation pairs** | **No. of Down-regulation pairs** | **No. of pairs (all)** |
| --- | --- | --- | --- | --- |
| small | train | 7,959 | 7,940 | 15,899 |
| small | valid | 1,016 | 971 | 1,987 |
| small | test | 962 | 1,026 | 1,988 |
| large | train | 12,151 | 12,101 | 24,252 |
| large | valid | 1,503 | 1,529 | 3,032 |
| large | test | 1,534 | 1,298 | 3,032 |

Table S2. Training details of Methven.

| **Model size** | **BiGRU layers** | **BiGRU units** | **Training GPU** | **Batch size** | **No. of model hypermeters** |
| --- | --- | --- | --- | --- | --- |
| small | 2 | 64 | 1*Nvidia 3090 | 1,024 | 722,786 |
| large | 2 | 64 | 1* Nvidia 3090 | 512 | 814,946 |

Table S3. Ablation study of Methven-small model in classification.

| **Model** | **ACC** | **Precision** | **Recall** | **F1-score** | **AUC** | **No. of model hypermeters** |
| --- | --- | --- | --- | --- | --- | --- |
| Without ATAC-seq | 0.8828 | 0.8777 | 0.8805 | 0.8791 | 0.9389 | 722,402 |
| Without DNABert2 | 0.5000 | 0.4891 | 0.7672 | 0.5973 | 0.5035 | 132,962 |
| Replace DNABert2 with One-Hot | 0.9095 | 0.8924 | 0.8920 | 0.9070 | 0.9548 | 5,245,794 |
| Replace BiGRUs with FC layers | 0.8778 | 0.8772 | 0.8690 | 0.8731 | 0.9424 | 126,882 |
| Methven-small | 0.9120 | 0.9174 | 0.9063 | 0.9116 | 0.9654 | 722,786 |

Table S4. Ablation study of Methven-large model in classification.

| **Model** | **ACC** | **Precision** | **Recall** | **F1-score** | **AUC** | **No. of model hypermeters** |
| --- | --- | --- | --- | --- | --- | --- |
| Without ATAC-seq | 0.8087 | 0.8210 | 0.7953 | 0.8079 | 0.8872 | 814,562 |
| Without DNABert2 | 0.5129 | 0.5098 | 0.7761 | 0.6474 | 0.5700 | 225,122 |
| Replace DNABert2 with One-Hot | 0.8174 | 0.8269 | 0.8277 | 0.8248 | 0.9065 | 51,325,794 |
| Replace BiGRUs with FC layers | 0.8183 | 0.8377 | 0.7705 | 0.8027 | 0.8935 | 219,042 |
| Methven-large | 0.8480 | 0.8463 | 0.8530 | 0.8490 | 0.9271 | 814,946 |

Table S5. Comparison of existing methods in classification.

| **Model size** | **Model** | **ACC** | **Precision** | **Recall** | **F1-score** | **AUC** |
| --- | --- | --- | --- | --- | --- | --- |
| small | Methven | 0.9077±0.0091 | 0.9090±0.0090 | 0.9063±0.0145 | 0.9076±0.0093 | 0.9077±0.0091 |
|  | CpGenie* | 0.7818±0.1902 | 0.7399±0.2975 | 0.8261±0.2796 | 0.7656±0.2730 | 0.8067±0.2063 |
|  | Enformer | 0.8127±0.0122 | 0.8141±0.0173 | 0.8116±0.0144 | 0.8125±0.0129 | 0.8127±0.0122 |
| large | Methven | 0.8416±0.0089 | 0.8406±0.0122 | 0.8441±0.0058 | 0.8424±0.0073 | 0.8417±0.0089 |
|  | CpGenie* | 0.7057±0.1706 | 0.6564±0.2670 | 0.8076±0.2780 | 0.7071±0.2486 | 0.7388±0.1975 |
|  | Enformer | 0.7554±0.0088 | 0.7544±0.0149 | 0.7593±0.0135 | 0.7568±0.0100 | 0.7555±0.0088 |

* Predictions beyond the scope of CpGenie use a retrained CpGenie model.

Table S6. External analysis on monocyte meQTLs.

| **Model size** | **Model** | **ACC** | **Precision** | **Recall** | **F1-score** | **AUC** |
| --- | --- | --- | --- | --- | --- | --- |
| small | end-to-end | 0.845 | 0.802 | 0.890 | 0.843 | 0.898 |
|  | fine-tune | 0.888 | 0.868 | 0.898 | 0.883 | 0.939 |
| large | end-to-end | 0.704 | 0.665 | 0.825 | 0.736 | 0.770 |
|  | fine-tune | 0.763 | 0.742 | 0.809 | 0.774 | 0.823 |

Table S7. Prediction results of Methven-small on rs968567 (chr11: 61595564) in disease-SNP analysis.

| **CpG site** | **CpG position** | **CpG-SNP distance** | **Slope(0h)** | **Slope(24h)** |
| --- | --- | --- | --- | --- |
| cg07709195 | 61586015 | 9,549 | 3.30768 | 3.107404 |
| cg08281583 | 61595223 | 341 | -0.97285 | 0.414493 |
| cg02563962 | 61595550 | 14 | 0.895135 | -0.17976 |
| cg20896974 | 61595983 | 419 | 1.704718 | 0.675927 |
| cg15454066 | 61595377 | 187 | -0.55628 | -0.67076 |
| cg21409469 | 61594769 | 795 | -1.50895 | 0.143971 |
| cg07005513 | 61595956 | 392 | 1.896686 | 1.641777 |
| cg19481605 | 61596812 | 1,248 | -2.00758 | -0.96298 |
| cg06781209 | 61594997 | 567 | -1.18766 | 0.085661 |
| cg14911132 | 61596755 | 1,191 | 2.173507 | 3.008933 |
| cg05698098 | 61595494 | 70 | -1.24906 | -1.56824 |
| cg27386326 | 61587980 | 7,584 | -3.43851 | -2.88585 |
| cg25324164 | 61598330 | 2,766 | 0.943897 | -0.18036 |
| cg13299762 | 61594708 | 856 | -2.75586 | -1.79238 |
| cg08380661 | 61592206 | 3,358 | 2.004973 | 1.569955 |
| cg07999042 | 61598930 | 3,366 | -2.5631 | -2.4589 |
| cg23760165 | 61595485 | 79 | -0.09937 | -0.28168 |
| cg14562930 | 61595050 | 514 | -1.89603 | -1.14012 |
| cg05816884 | 61595492 | 72 | -0.91971 | -0.94432 |
| cg19610905 | 61596333 | 769 | 0.973409 | -0.29367 |
| cg08093537 | 61595225 | 339 | -0.29462 | 0.866731 |
| cg25303599 | 61595807 | 243 | 1.69671 | 1.639525 |
| cg00614641 | 61596405 | 841 | -2.71884 | -3.82376 |
| cg10515671 | 61585899 | 9,665 | 1.785255 | 1.755254 |
| cg09610223 | 61595465 | 99 | -0.13369 | -0.34711 |
| cg22796604 | 61602232 | 6,668 | -3.07965 | -3.84196 |
| cg00603274 | 61596626 | 1,062 | 0.998823 | 1.712455 |
| cg21709803 | 61594965 | 599 | -2.26192 | -0.98825 |
| cg11250194 | 61601937 | 6,373 | -2.95164 | -2.51638 |
| cg10868875 | 61596307 | 743 | -0.01807 | -1.01722 |
| cg21029357 | 61601062 | 5,498 | -1.52554 | -1.73847 |
| cg01400685 | 61598025 | 2,461 | 1.165036 | -0.20022 |

Table S8. The data distribution of the external monocyte dataset.

| **Model size** | **type** | **No. of Up-regulation pairs** | **No. of Down-regulation pairs** | **No. of pairs (all)** |
| --- | --- | --- | --- | --- |
| small | train | 2,014 | 2,000 | 4,014 |
| small | valid | 259 | 243 | 502 |
| small | test | 236 | 266 | 502 |
| large | train | 3,371 | 3,377 | 6,748 |
| large | valid | 420 | 423 | 843 |
| large | test | 423 | 421 | 844 |

Table S9. Comparison between DNA language models in the regression task. For the fairness of comparison, we use three tree models that can obtain the optimal parameters by grid search, and obtain the optimal parameters by grid search. The dataset used pairs with SNP-CpG distances of less than 10kbp in the Methven training set.

| **Embedding** | **Model** | **RMSE** | **R-square** | **PCC** | **PCC P-value** |
| --- | --- | --- | --- | --- | --- |
| DNAbert2 | LightGBM | 2.242 | 0.517 | 0.722 | 2.530×10^-65^ |
|  | XGBoost | 2.191 | 0.538 | 0.742 | 9.963×10^-71^ |
|  | Random Forest | 2.238 | 0.474 | 0.721 | 5.766×10^-65^ |
| Hyena | LightGBM | 2.138 | 0.560 | 0.751 | 4.059×10^-73^ |
|  | XGBoost | 2.191 | 0.538 | 0.742 | 9.963×10^-71^ |
|  | Random Forest | 2.337 | 0.474 | 0.720 | 9.564×10^-65^ |
| NT | LightGBM | 2.176 | 0.544 | 0.740 | 4.548×10^-70^ |
|  | XGBoost | 2.203 | 0.533 | 0.738 | 1.590×10^-69^ |
|  | Random Forest | 2.297 | 0.492 | 0.726 | 2.476×10^-66^ |

# Figures

Figure S1. The data distribution of the Methven intra-data in pre-processing progress.


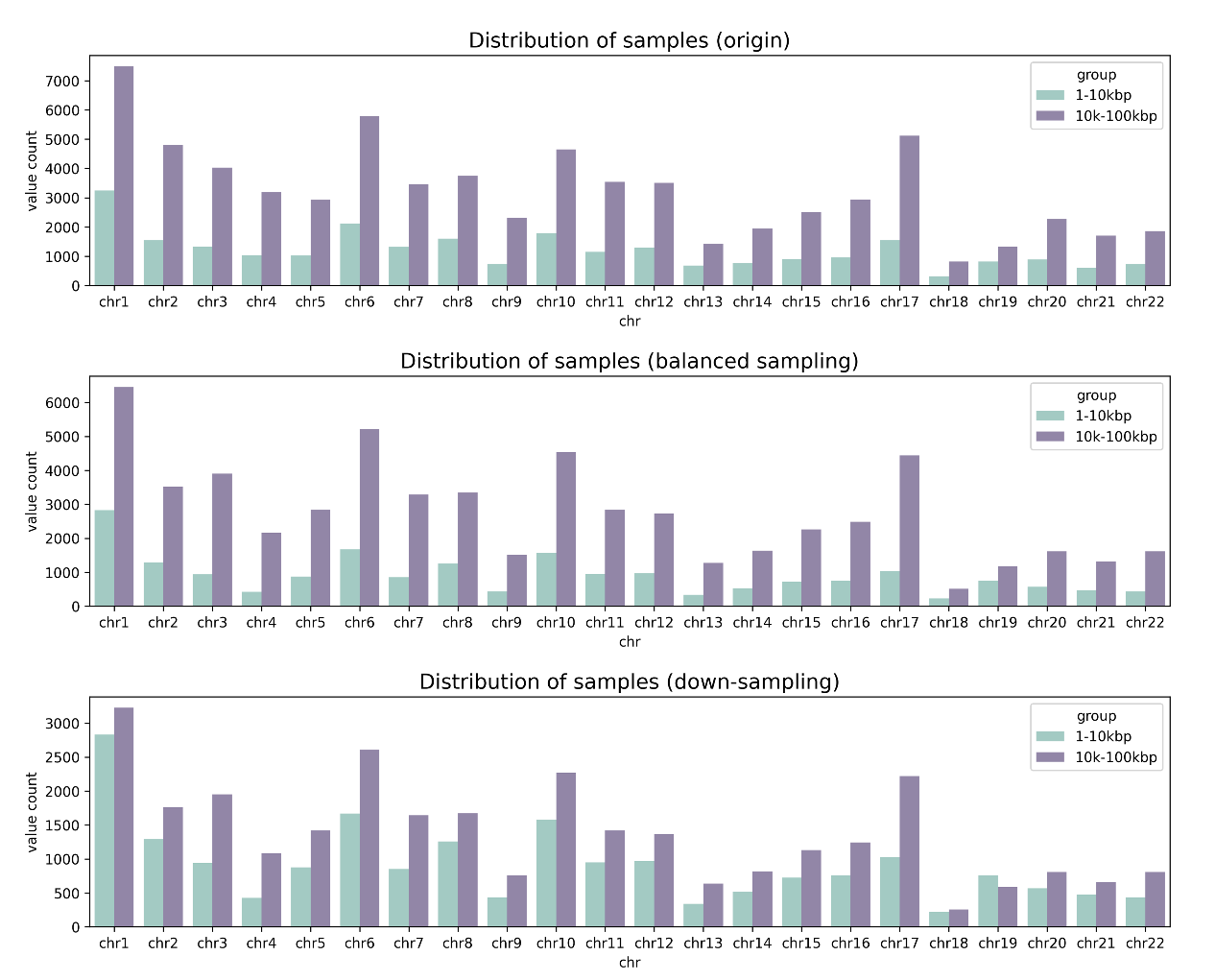


Figure S2. The data distribution of the external monocyte dataset in pre-processing progress.


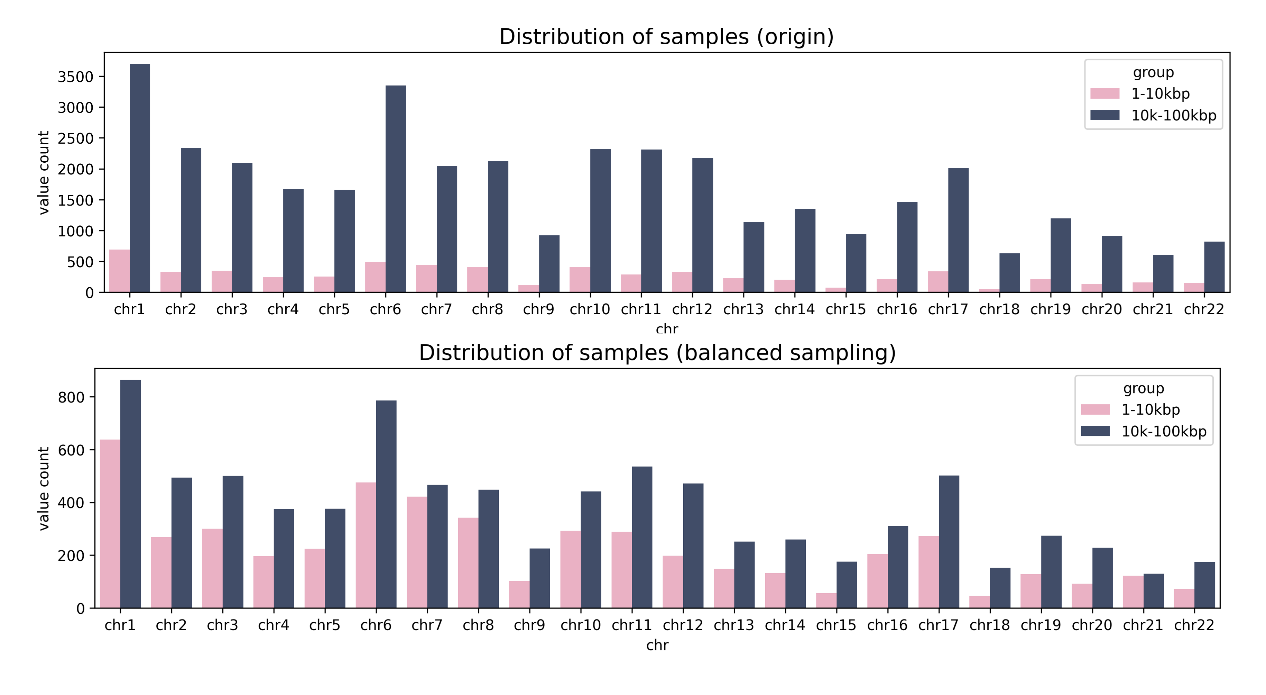


Figure S3. The performance of existing methods in model comparison.


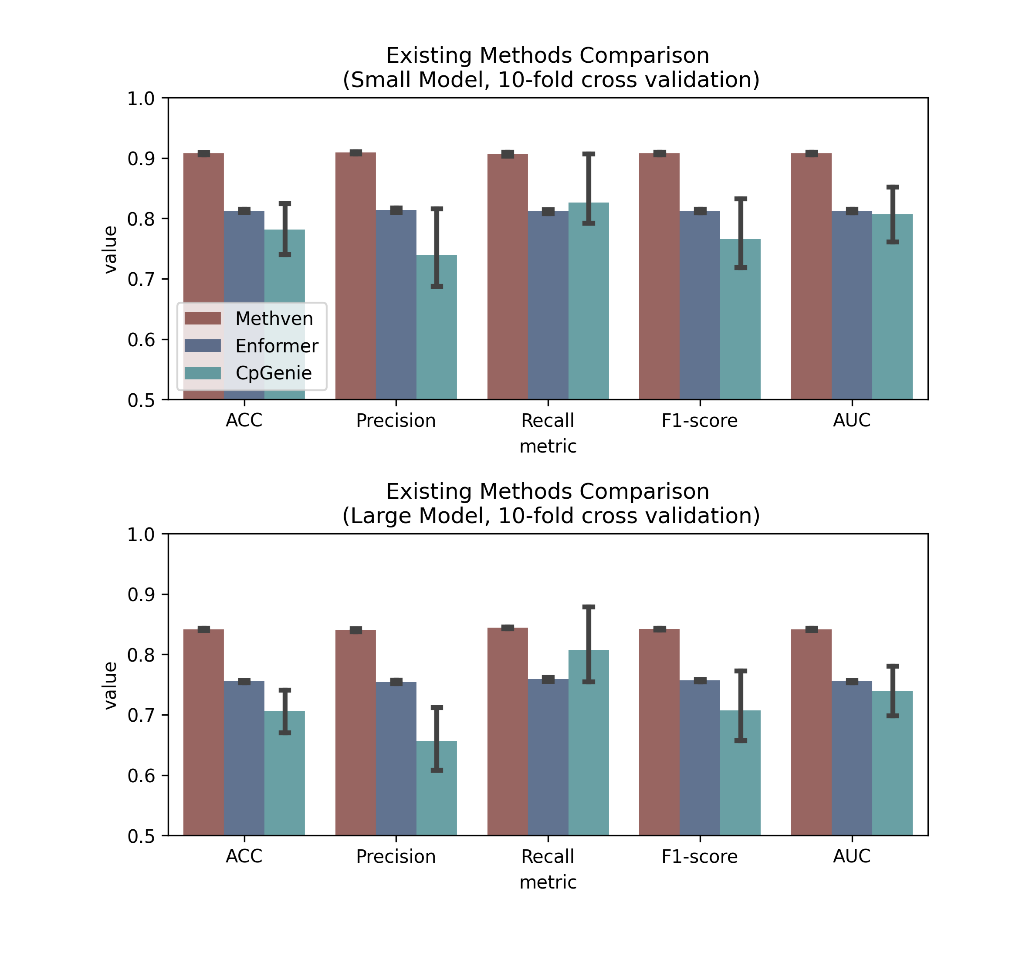


Figure S4. Preliminary evaluation of Methven’s generalizability to tissue-level meQTLs using a small-scale retina meQTL dataset. The retina meQTL dataset was downloaded from https://zenodo.org/records/10569726 and the corresponding tissue ATAC-seq data was downloaded from EpiMap Repository (BSS number: BSS01502). Following data preprocessing and down-sampling, we created a small dataset containing 2,602 meQTLs, which we split into training, validation, and test sets in an 8:1:1 ratio.


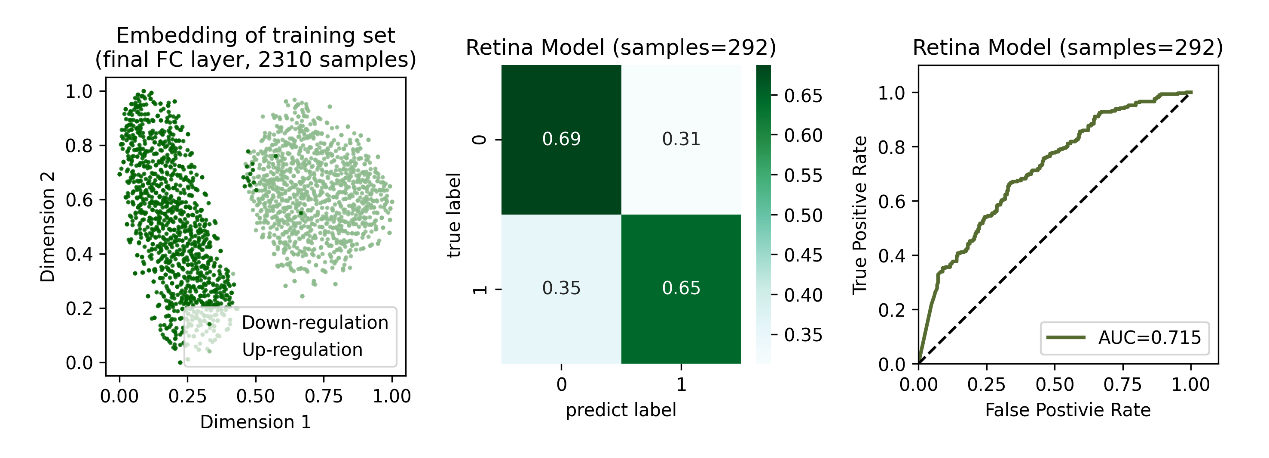


Figure S5. Extending Methven to predict the effect of mutations on gene expression (eQTL prediction). The B naive, CD4 memory, and CD8 memory single-cell eQTL datasets were obtained from the OneK1K cohort, with the corresponding ATAC-seq data downloaded from EpiMap Repository (BSS numbers: BSS00097, BSS00185, BSS00193). After preprocessing and down-sampling, the datasets consisted of 1,878, 3,544, and 2,306 eQTLs, respectively. Each eQTL was annotated with TSS locations using hgTables, and the input positions for Methven were modified to center on TSS regions instead of CpG sites while ensuring that the SNPs were within the input window.


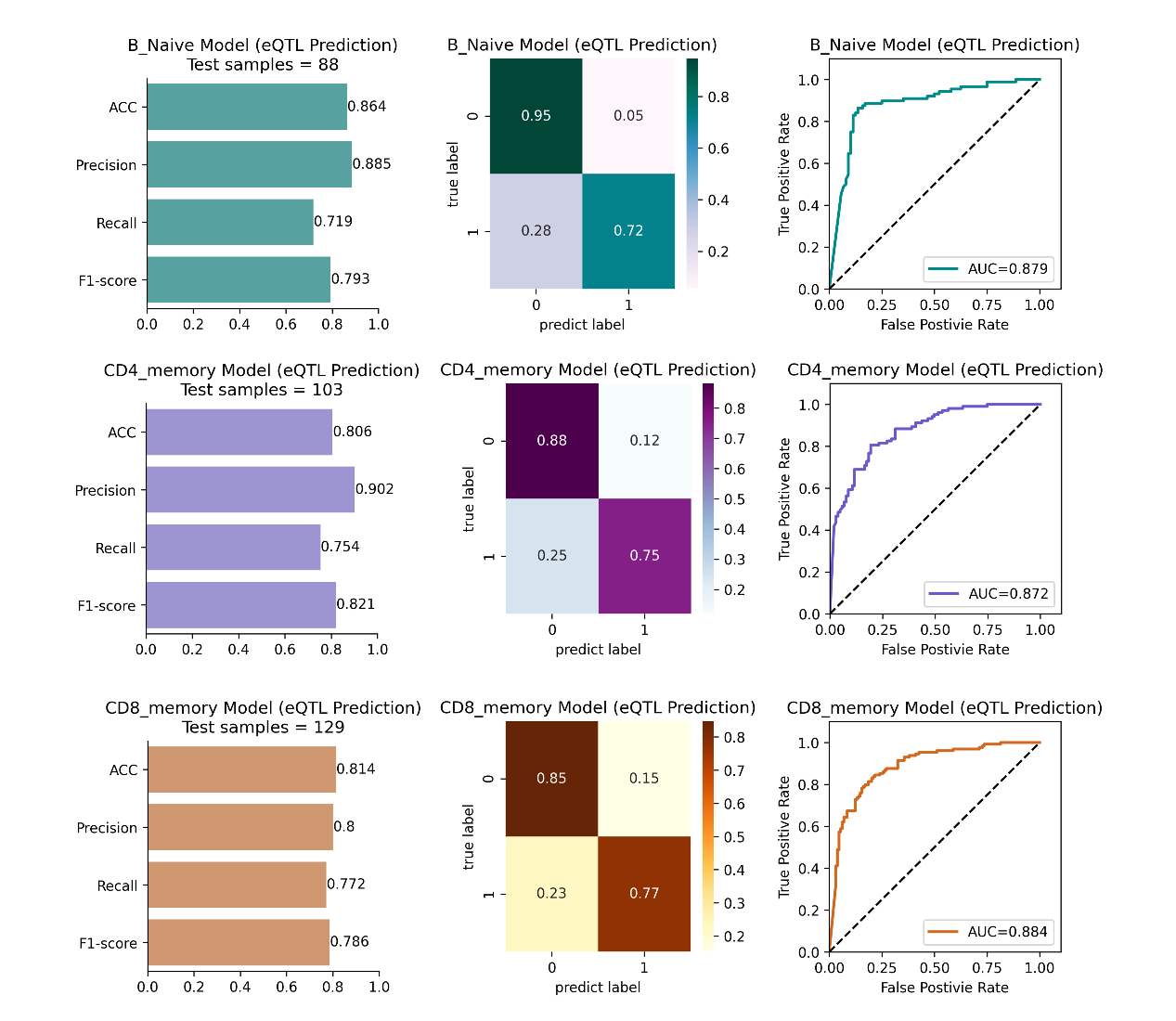


Figure S6. Comparison between DNA language models in the regression task. For the fairness of comparison, we use three tree models that can obtain the optimal parameters by grid search, and obtain the optimal parameters by grid search. The dataset used pairs with SNP-CpG distances of less than 10kbp in the Methven training set.


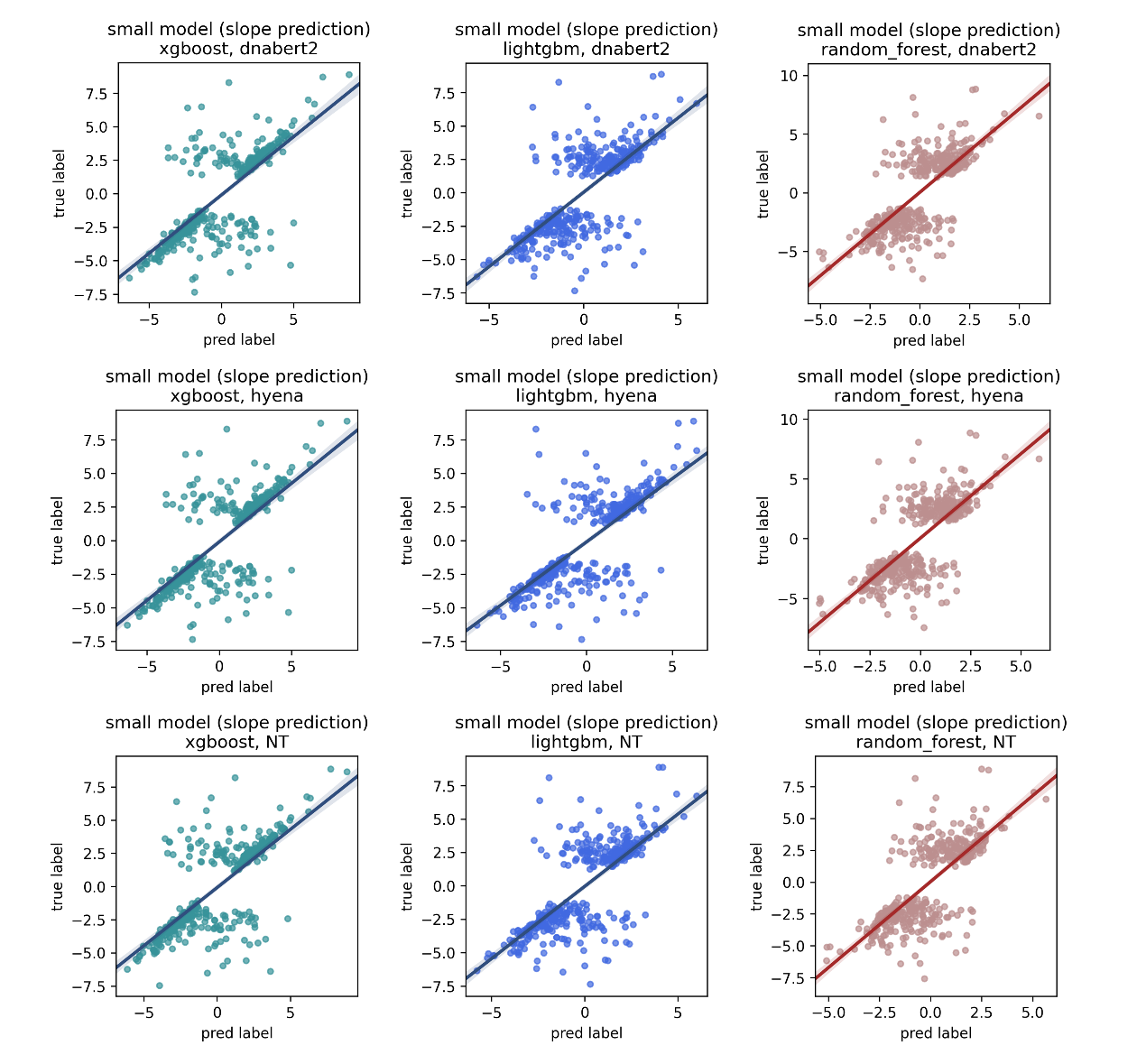

Supplement: Supplementary file 1 — Supporting Information [file ADVS-12-2413571-s001.docx]
